# Mapping Perfusion and Predicting Success: Infrared Thermography-Guided Perforator Flaps for Lower Limb Defects

**DOI:** 10.3390/medicina61081410

**Published:** 2025-08-03

**Authors:** Abdalah Abu-Baker, Andrada-Elena Ţigăran, Teodora Timofan, Daniela-Elena Ion, Daniela-Elena Gheoca-Mutu, Adelaida Avino, Cristina-Nicoleta Marina, Adrian Daniel Tulin, Laura Raducu, Radu-Cristian Jecan

**Affiliations:** 1 Doctoral School, “Carol Davila” University of Medicine and Pharmacy, 010221 Bucharest, Romania; abdalah.abu-baker@drd.umfcd.ro; 2 Discipline of Plastic Surgery, “Carol Davila” University of Medicine and Pharmacy, 010221 Bucharest, Romania; andrada-elena.tigaran@rez.umfcd.ro (A.-E.Ţ.); teodora.peligrad@rez.umfcd.ro (T.T.); ion.daniela1@gmail.com (D.-E.I.); adelaida.avino@gmail.com (A.A.); cristina.cozma88@gmail.com (C.-N.M.); jecan.radu@gmail.com (R.-C.J.); 3 Department of Plastic Surgery, “Prof. Dr. Agrippa Ionescu” Emergency Clinical Hospital, 011356 Bucharest, Romania; daniela-elena.mutu@umfcd.ro; 4 Discipline of Anatomy, “Carol Davila” University of Medicine and Pharmacy, 010221 Bucharest, Romania; 5 Department of General Surgery, “Prof. Dr. Agrippa Ionescu” Emergency Clinical Hospital, 011356 Bucharest, Romania

**Keywords:** infrared thermography, Doppler ultrasonography, perforator flaps, lower limb reconstruction, reconstructive surgery, flap planning, flap metrics

## Abstract

*Background and Objectives*: Lower limb defects often present significant reconstructive challenges due to limited soft tissue availability and exposure of critical structures. Perforator-based flaps offer reliable solutions, with minimal donor site morbidity. This study aimed to evaluate the efficacy of infrared thermography (IRT) in preoperative planning and postoperative monitoring of perforator-based flaps, assessing its accuracy in identifying perforators, predicting complications, and optimizing outcomes. *Materials and Methods*: A prospective observational study was conducted on 76 patients undergoing lower limb reconstruction with fascio-cutaneous perforator flaps between 2022 and 2024. Perforator mapping was performed concurrently with IRT and Doppler ultrasonography (D-US), with intraoperative confirmation. Flap design variables and systemic parameters were recorded. Postoperative monitoring employed thermal imaging on days 1 and 7. Outcomes were correlated with thermal, anatomical, and systemic factors using statistical analyses, including *t*-tests and Pearson correlation. *Results*: IRT showed high sensitivity (97.4%) and positive predictive value (96.8%) for perforator detection. A total of nine minor complications occurred, predominantly in patients with diabetes mellitus and/or elevated glycemia (*p* = 0.05). Larger flap-to-defect ratios (A/C and B/C) correlated with increased complications in propeller flaps, while smaller ratios posed risks for V-Y and Keystone flaps. Thermal analysis indicated significantly lower flap temperatures and greater temperature gradients in flaps with complications by postoperative day 7 (*p* < 0.05). CRP levels correlated with glycemia and white blood cell counts, highlighting systemic inflammation’s impact on outcomes. *Conclusions*: IRT proves to be a reliable, non-invasive method for perforator localization and flap monitoring, enhancing surgical planning and early complication detection. Combined with D-US, it improves perforator selection and perfusion assessment. Thermographic parameters, systemic factors, and flap design metrics collectively predict flap viability. Integration of IRT into surgical workflows offers a cost-effective tool for optimizing reconstructive outcomes in lower limb surgery.

## 1. Introduction

Traumatic injuries are the most common cause of soft tissue loss in the lower limbs. Other causes include tumor resections, diabetic and vascular disease, and soft tissue infections. Due to the reduced soft tissue mobility in the lower limb, especially in the calf and foot, extensive defects at this level cannot be closed through direct suture, thus requiring the utilization of more complex reconstructive methods [1]. Skin grafts provide an accessible and fast reconstructive solution, but in turn cannot properly shield functional structures such as nerves, tendons, and deperiosted bone. Moreover, skin grafts may lead to recipient site morbidity when used in large and deep defects [2].

Flaps are currently considered a reliable option in treating lower limb defects, with the advantage of replacing the missing tissue with a substitute with similar characteristics (“like with like” flap coverage) [3]. The evolution of flap elevation techniques has progressed from the use of random-pattern flaps to muscle flaps, and subsequently to perforator-based local and free flaps [4]. A pivotal contribution to this advancement is Taylor’s “perforasome” concept, which has become a foundational principle in the field of flap surgery [5]. According to his principle, every area of skin can be assigned a perforator pedicle, usually comprised of one artery and one or two veins, which supply the respective patch [6]. Several anatomical studies showed that perforators are mostly clustered, and their location is fairly constant and easily predictable [5,7,8,9]. Thus, perforator vessels can be successfully used in elevating fascio-cutaneous flaps, greatly enlarging the plastic surgeon’s arsenal. Perforator-based advancement and rotation flaps—such as the keystone flap or the propeller flap—have been demonstrated to be dependable, reasonably quick solutions, capable of producing an aesthetically satisfying result, with no injury to the main artery and little donor site morbidity [10].

Infrared thermography (IRT) is a non-invasive technique that provides data on variations in body temperature caused by a range of situations, including physical activity, metabolic changes, rheumatic diseases, tumors, and musculoskeletal disorders [11,12]. These variations in skin temperature are indicative of underlying processes and subtle changes in capillary blood flow, with inflammation leading to a rise in thermal readings, whilst thickening of the hypodermis decreases the local temperature [12,13].

The operating principle of IRT revolves around infrared radiation, which has longer wavelengths than visible light and is located below red in the electromagnetic spectrum [14]. Every object or surface with an absolute temperature greater than 0 Kelvin releases infrared radiation. This kind of emission is invisible to the human eye; thus, infrared measurement tools are required to gather and evaluate this data [15]. Infrared cameras pick up the radiation transmitted by the subject and convert it into an electronic signal. By assigning each infrared energy level a color, frames captured by infrared cameras are transformed into color-coded images, called thermograms [16]. Thus, IRT provides a quick, painless, non-invasive, non-contact, economical, and radiation-free way to display temperature variations on the skin’s surface in an easily interpretable solution [17].

When performing perforator flap surgery, adequate tissue perfusion is crucial. The location and condition of perforators can be determined by preoperative imaging. Such data can shorten operating times and streamline the surgical process [18]. Infrared thermography has emerged as a valuable non-invasive tool in flap planning and postoperative monitoring. IRT uses skin rewarming patterns to assess dermal perfusion, thus revealing perforator location during the flap planning stage [19,20]. Moreover, IRT allows for real-time observation of cutaneous heat distribution, which aids in assessing perfusion dynamics and early diagnosis of vascular compromise. These factors contribute to enhanced surgical precision and patient outcomes.

This study aims to evaluate the effectiveness of IRT in preoperative planning and postoperative assessment of perforator-based flaps for lower limb reconstruction. Specifically, it seeks to determine the reliability of IRT in identifying perforator locations, assessing tissue perfusion, and optimizing surgical outcomes, while also evaluating its use in predicting flap complications. Our research intends to support the hypothesis that IRT improves perforator detection and vascular injury prediction through early thermographic changes, thereby enhancing surgical planning and outcomes in lower limb reconstruction.

## 2. Materials and Methods

A prospective observational study was designed to assess the efficacy of thermal imaging in the preoperative planning and postoperative monitoring of lower limb flaps, as well as to evaluate the influence of various systemic factors on flap outcomes. The study cohort comprised 76 patients admitted to the Plastic Surgery Department of “Prof. Dr. Agrippa Ionescu” Emergency Clinical Hospital between 1 January 2022, and 31 December 2024, with lower limb soft tissue defects requiring reconstruction using fascio-cutaneous perforator flaps.

Inclusion criteria:

- Patients with lower-limb defects treated using perforator flap coverage;
- Adults (at least 18 years old);
- Patients who agreed to be included in the study.

Exclusion criteria:

- Underage patients (younger than 18 years old);
- Pregnant women (pregnancies were noted based on patient history);
- Patients who refused to participate (1 patient);
- Patients discharged earlier than one week postoperatively (5 patients).

We systematically monitored multiple variables, categorized as follows:

- Patient-dependent factors: Age, gender, presence of diabetes, smoking status (patient considered a smoker if they smoked every day for at least a year).
- Flap-specific parameters: Number and location of perforators, flap type and size, absolute and relative flap temperatures recorded on postoperative day one and seven, and occurrence of flap-related complications.
- Paraclinical data: hemoglobin, leukocytes, glycemia, and C-reactive protein (CRP) levels prior to flap coverage.

**Figure 1:**
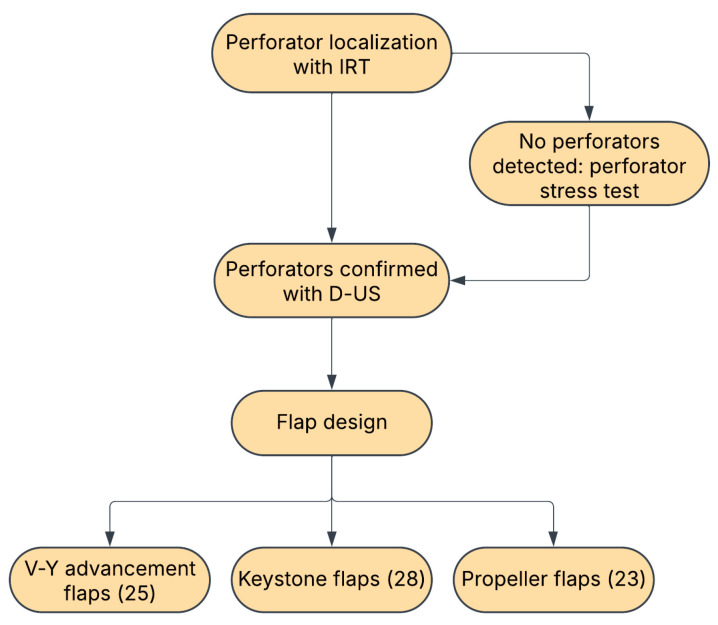
Perforator mapping and flap design protocol.

During the preoperative planning phase, perforator vessels adjacent to the defect were identified using a FLIR^®^ E50 Infrared Thermal Imaging Camera (Teledyne, Thousand Oaks, CA, USA). Perforators appeared as localized hotspots on thermal images and were subsequently marked. In cases where perforators were not readily detectable, a perforator stress test was performed to enhance visualization of arterial blood flow to the skin. This test involved applying 70% isopropyl alcohol to the target area and assessing temperature variations using IRT. The alcohol acted as a rapid cooling agent, facilitating perforator localization by revealing early rewarming hotspots. Once marked, perforator locations were further validated using a LifeDop^®^ L250 Handheld Doppler Ultrasound Device (Cooper Surgical, Trumbull, CT, USA) equipped with an 8 MHz surface probe. Only perforators located with IRT and confirmed with Doppler ultrasonography (D-US) were used in flap design. Based on the number and location of available perforators, and taking into consideration the particularities of the defect and surrounding tissues, perforator-based flaps were designed using one of three techniques: V-Y advancement flaps (25 cases), Keystone flaps (28 cases), or Propeller flaps (23 cases). The summarized flap design protocol is described in Figure 1.

**Figure 2:**
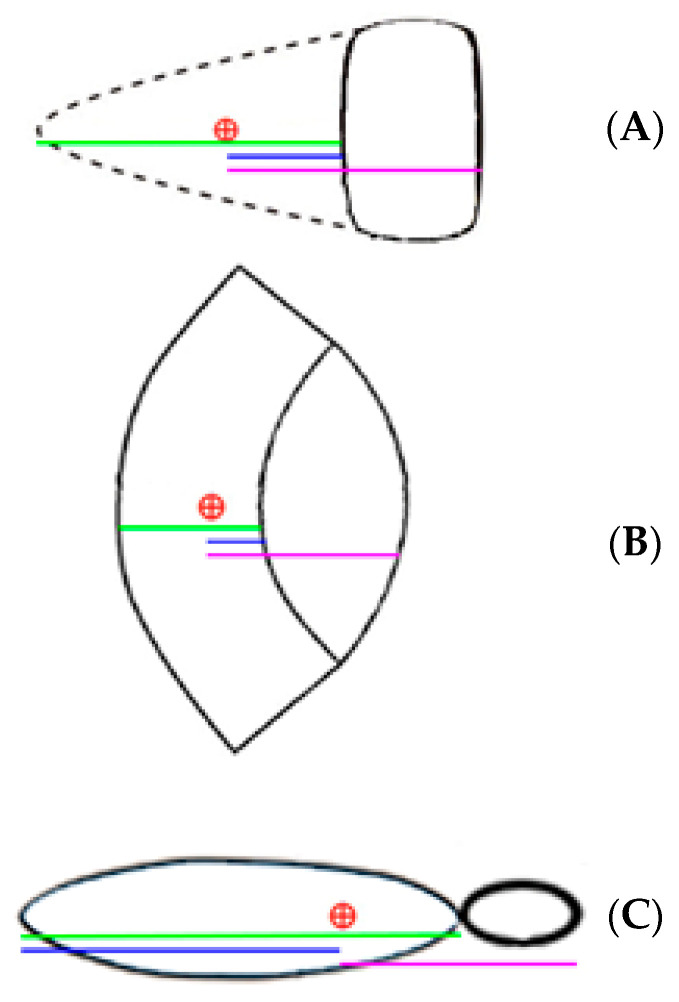
Red cross-mark = perforator location; green segment = flap length (**A**); blue segment = perforator-to-flap-edge distance (**B**); pink segment = perforator-to-defect-edge distance (**C**).

During flap design, we defined and noted three flap-dependent variables (as exemplified in Figure 2):

- Flap length (A) = the total length of the flap corresponding to the diameter that runs through the defect’s center.
- Perforator-to-flap-edge distance (B) = the distance between the flap edge that is transposed across the defect and the chosen flap-supplying perforator. Note that, in the cases of V-Y and keystone flaps, the flap edge overlaying the defect is oriented towards the defect, while for propeller flaps, the involved edge is oriented away from the defect.
- Perforator-to-defect-edge distance (C) = the distance from the chosen flap-supplying perforator to the opposite defect edge.

Preoperatively, flap design was customized based on the anatomical and vascular particularities of the segment to be reconstructed, starting with distance C, which is predetermined by the defect size and perforator location. For V-Y advancement flaps and Keystone flaps, the flap length (A) was chosen to be 0.5 to 1.0 cm longer than the C distance, in order to ensure adequate tissue mobilization and tension-free closure. On the other hand, for propeller flaps, the distance (B) between the pivot point and the flap’s distal tip was designed to be 0.5–1.0 cm greater than the C distance, thus ensuring sufficient arc of rotation and skin paddle in order to cover the defect.

Each procedure started with either wound debridement or tumor resection, followed by evaluation of the defect, identification of exposed structures, and intraoperative reassessment of surrounding tissue mobility. After adjusting the design of the flap, skin incisions were carried out according to the intraoperative drawing. Dissection was performed in the suprafascial plane. After flap elevation, the perforator vessels were meticulously dissected, documented, and their blood flow was assessed using Doppler ultrasound (D-US) to evaluate patency and ensure adequate perfusion of the skin paddle.

In the postoperative period, all patients received standardized management, including daily dressing changes and clinical evaluation. To further assess flap viability, IRT was utilized at two predefined time points: postoperative day one and postoperative day seven. Absolute temperature measurements were obtained by identifying the brightest hotspot on the thermal image of the flap. Relative temperature measurements were calculated by subtracting the flap temperature from the mean temperature of the surrounding skin, providing an additional parameter for perfusion assessment.

The data used in this study were gathered using Microsoft Office Excel 2021. Statistical analysis was conducted using SPSS software, version 26.0. A range of statistical tests were used to evaluate the collected data. Specifically, Chi-square (χ^2^) tests were employed to analyze associations between categorical variables, independent samples *t*-tests were applied to compare means between groups for continuous variables, and Pearson’s correlations were used to examine the strength and direction of linear relationships between continuous variables. A 95% confidence interval was applied to all statistical tests. The manuscript was drafted and edited using Microsoft Office Word 2021.

## 3. Results

The study group was comprised of 76 patients, of which 45 (59.2%) were males and 31 (40.7%) were females. The patients ranged from 20 years old to 83 years old, with a mean age of 59 years. Among the observed population, we encountered 24 (31.5%) patients suffering from type II diabetes mellitus and 39 (51.3%) active tobacco users. Moreover, 22 (28.9%) patients in our study group were both diabetics and active smokers at the time of the study.

Throughout the study, no cases of major flap failure were observed. However, minor flap complications occurred, including six cases (7.8%) of peripheral wound dehiscence and three cases (3.9%) of superficial flap necrosis. All complications were managed conservatively with daily dressing changes, leading to successful healing through secondary epithelialization. Notably, no flaps required surgical reintervention.

### 3.1. Influence of FLAP Design on Complications

After confirming perforator locations, flap design was initiated by first measuring the distance from the perforator to the defect. For V-Y advancement flaps and Keystone flaps, this measurement represents the B dimension. Subsequently, the C measurement was determined by measuring the distance from the same perforator to the furthest edge of the defect, which guided the required flap length.

Flap design was tailored accordingly:

- For V-Y advancement and Keystone flaps, the flap length (A) was designed to be 0.5 cm to 1 cm longer than the C distance.
- For Propeller flaps, the B distance was selected to be 0.5 cm to 1 cm longer than the C distance.

**Figure 3:**
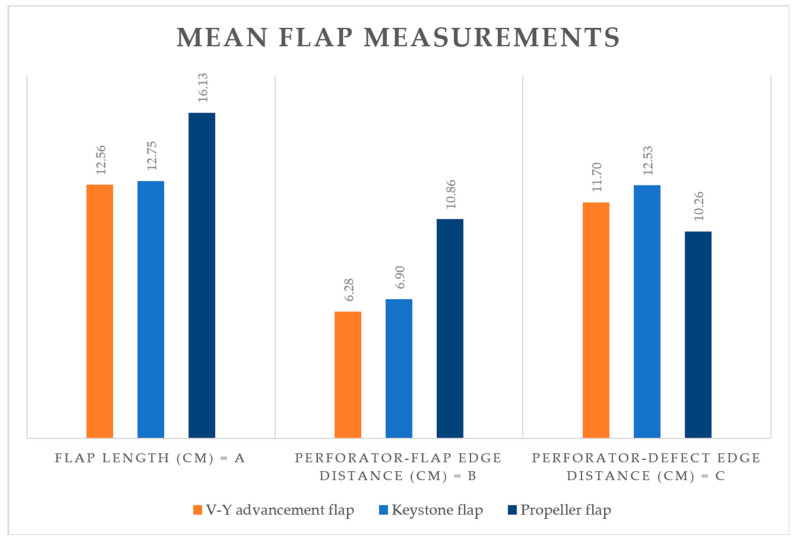
Mean flap measurements depending on flap type.

Descriptive data regarding flap measurements are provided in Table 1 and illustrated in Figure 3.

The differences in flap measurements reflect the variability in flap design methodology, with propeller flaps tending to be longer while the perforator-to-defect distance is shorter.

To systematically evaluate the impact of flap design on postoperative outcomes, we calculated two flap-dependent ratios based on the previously defined measurements. The perforator–defect distance was selected as the reference scale, as this dimension is independent of the flap design approach:

- Total flap length/perforator–defect distance (A/C);
- Perforator–flap edge distance/perforator–defect distance (B/C).

Due to the inherent differences in flap planning methodologies, a direct comparison between flap types was not feasible, necessitating separate data analyses. The previously defined ratios (A/C and B/C) were evaluated using independent *t*-tests to assess their relationship with postoperative complications.

The nine cases of minor complications observed in the study cohort were distributed as follows:

- V-Y advancement flaps: two cases (8%);
- Keystone flaps: two cases (7.1%);
- Propeller flaps: five cases (21%).

A higher B/C or A/C ratio generally indicates a larger flap relative to the defect, whereas lower ratios correspond to smaller flaps in proportion to the defect size. Data analysis suggests that lower ratios were associated with higher complication rates in V-Y advancement and Keystone flaps, likely due to excessive flap advancement and increased suture tension at flap margins. However, the limited sample size resulted in a lack of statistical significance for these flap types (Table 2).

Conversely, for propeller flaps, complications were more prevalent with higher A/C and B/C ratios, indicating that larger flaps were more prone to unfavorable outcomes, potentially due to perfusion-related issues. This finding was statistically significant (t(21) = −2.2, *p* = 0.037).

### 3.2. Non-Invasive Detection of Cutaneous Perforators and Implications on Flap Outcome

In this study, perforators were detected and documented transcutaneously using infrared thermography (IRT) and Doppler ultrasound (D-US), and their location and patency were confirmed intraoperatively. Each detection technique identified between 1 and 4 perforators, with the following average number of perforators detected per flap:

- First technique: 2.07 perforators/flap using IRT;
- Second technique: 1.87 perforators/flap using D-US;
- Third technique: 2.05 perforators/flap through direct intraoperative observation.

The number of perforators detected by each method is presented in Table 3.

The sensitivity and positive predictive value of each detection method relative to intraoperative observation were calculated. The results were as follows:

- IRT (Infrared Thermography): sensitivity = 97.4%; positive predictive value = 96.8%;
- D-US (Doppler Ultrasound): sensitivity = 90.3%; positive predictive value = 99.2%.

These findings indicate that IRT is a highly efficient method for detecting perforators, while D-US provides additional value as a validation technique.

When analyzing the impact of detected perforators on flap outcomes, our data suggest that fewer perforators are associated with adverse outcomes, with statistical significance, as shown in Table 4. Specific details are presented below:

- IRT identified a mean of 1.53 perforators in flaps that experienced postoperative complications, compared to 2.13 perforators in flaps with optimal healing (t(74) = 2.067, *p* = 0.042).
- D-US identified a mean of 1.33 perforators in flaps with complications, versus 1.94 perforators in those without complications (t(74) = 2.332, *p* = 0.022).

Essentially, intraoperative detection of perforators had the greatest impact on flap outcomes, with a mean of 1.33 perforators identified in flaps with complications, compared to 2.15 perforators in those with uneventful recovery (t(74) = 2.899, *p* = 0.005).

### 3.3. Implications of Systemic Variables

In evaluating systemic factors with an influence on flap integration, we analyzed multiple variables that are known to impair peripheral blood supply. Among the nine cases of minor complications, all patients suffered from diabetes mellitus, while 7 (77.8%) of these patients had a history of tobacco consumption (Table 5).

Statistical analysis revealed that diabetes mellitus had a significant impact on the complication rate (X^2^(1, N = 76) = 22.1, *p* < 0.001), with a moderate effect size (φ = 0.539). Although smoker status did not reach statistical significance, there was a clear trend suggesting the detrimental nature of this factor on flap outcomes.

Paraclinical data provide valuable insight into the general health status of patients before surgery. In this study, serum levels of hemoglobin (Hb), white blood cells (WBCs), glycemia, and C-reactive protein (CRP) were recorded to assess potential imbalances in preoperative homeostasis.

The results showed that hemoglobin levels did not significantly affect flap outcomes, while white blood cell (WBC) counts were slightly elevated in cases with complications compared to those with uneventful recoveries. CRP levels were notably higher in flaps with suboptimal healing, increasing by 225% compared to those without complications.

Glycemia had the most substantial impact on flap outcomes. The mean preoperative glucose level for compromised flaps was 181.22 mg/dl, compared to 116.42 mg/dl in the remaining cases (t(8.73) = −2.27, *p* = 0.05). Furthermore, the effect size for preoperative hyperglycemia was substantial, with Cohen’s D = −1.22, indicating a significant role of hyperglycemia in influencing flap success (Table 6).

Although no single paraclinical measurement can perfectly predict patient status, we observed that certain variables can provide insight into multiple physiological systems. Our data analysis revealed that CRP levels demonstrated statistically significant correlations (*p* < 0.001) with all other recorded serum parameters, exhibiting varying degrees of association:

- Hemoglobin (Hb): r(76) = −0.507;
- White blood cells (WBC): r(76) = 0.632;
- Glycemia: r(76) = 0.596.

These findings suggest that CRP levels may serve as a useful marker of systemic inflammation and correlate with other biological markers that reflect overall health status, which in turn impacts the postoperative evolution of the flap.

### 3.4. Efficacy of IRT Monitoring in Predicting Flap Outcomes

Flap temperature measurements revealed a mean temperature of 33.8 °C on the first postoperative day, which increased to 35.6 °C by the seventh day. Additionally, the mean flap temperature difference on the first day post-surgery was recorded at 3.1 °C, which decreased and normalized to 0.6 °C after 7 days.

When considering diabetic status, a statistically significant difference was observed, particularly at the seven-day postoperative threshold. Specifically, diabetic patients had lower flap temperatures (M = 35.04 °C) compared to nondiabetic patients (M = 35.85 °C) (t(26.359) = 4.256, *p* < 0.001), as shown in Table 7. Furthermore, the temperature gradient between the flap and surrounding tissue was greater in diabetic patients (M = 0.98 °C) compared to normoglycemic patients (M = 0.4 °C) when measured at the one-week timepoint (t(27.605) = −4.883, *p* < 0.001). These findings suggest that diabetic status has a notable impact on flap temperature dynamics and perfusion.

On the other hand, smoking had a substantial effect on thermal readings. Smokers exhibited colder flaps at both time points, with mean flap temperatures of 33.5 °C and 35.3 °C on the first and seventh postoperative days, respectively, compared to 34.1 °C and 35.9 °C for non-smokers (*p* < 0.001). The temperature gap between the flap and surrounding tissue was similar for smokers and non-smokers on the first postoperative day, as presented in Table 8. However, at one week after surgery, smokers showed a significantly higher temperature gap (M = 0.77 °C) compared to non-smokers (M = 0.39 °C) (t(74) = −3.9, *p* < 0.001). These findings suggest that smoking greatly influences perfusion, particularly as time progresses after surgery.

Regarding flap outcomes, we observed that lower temperatures were recorded for flaps with complications both on day one (33.4 °C vs. 33.8 °C, t(15.6) = 2.51, *p* = 0.023) and day seven (34.6 °C vs. 35.7 °C, t(8.5) = 3.01, *p* = 0.015) postoperatively, as indicated in Table 9.

**Figure 4:**
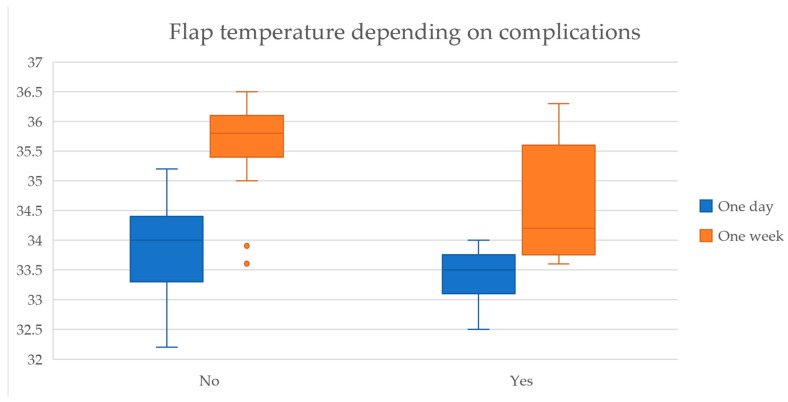
Distribution of mean flap temperatures depending on complication incidence.

Additionally, the temperature gradient between the flap and the surrounding skin was higher in complicated flaps at both time points, with a more pronounced difference at one-week post-surgery (1.19 °C vs. 0.51 °C, t(8.6) = −3.02, *p* = 0.015), shown in Figure 4.

Furthermore, the effect size of the thermal readings at the one-week measurement showed a high magnitude of association, with Cohen’s D coefficient of 1.84, suggesting that flap temperature plays a significant role in predicting postoperative outcomes.

## 4. Discussion

Soft-tissue coverage is paramount when limb salvage is taken into consideration, especially when dealing with cases of significant soft-tissue loss and exposure of essential structures. This study demonstrates that IRT is a highly effective, non-invasive aid in lower limb flap surgery, with high sensitivity (97.4%) in preoperative perforator detection and strong predictive value for postoperative complications based on temperature gradients. Key findings reveal that diabetic status, hyperglycemia, and flap geometry significantly influence outcomes, positioning IRT as a valuable, cost-effective adjunct in lower limb reconstructive surgery.

The development of biologic skin replacements and negative-pressure therapy has improved wound care and helped temporize wound management in the acute environment, providing a versatile bridging solution before definitive coverage can be achieved [21]. Nonetheless, locoregional flaps are a viable and successful option and represent a crucial technique towards soft tissue regeneration of the lower limb. Over the past 20 years, the value of locoregional flaps in lower limb soft tissue repair has grown significantly, thanks to major advancements in our understanding of the anatomy involved in these techniques [22]. Their strategic advantage lies in utilizing tissue with similar characteristics from adjacent regions, employing a robust vascular supply, thus facilitating superior integration and healing. Local flaps play an essential role in bridging the gap between complex wound defects and definitive reconstruction, particularly in cases where free tissue transfer may be contraindicated due to comorbidities, poor recipient vessel quality, or resource limitations. As such, they remain an indispensable component of the reconstructive surgeon’s armamentarium in lower limb salvage [23].

Multiple studies have demonstrated that it is generally safe to dissect fascio-cutaneous flaps within the territory between two adjacent perforators, irrespective of their anatomical localization. This concept is supported by a growing number of anatomical and clinical studies indicating that the interperforator region possesses sufficient perfusion to sustain flap viability. Even where the distance between perforators is relatively large, the vascular interconnections provide adequate blood flow to support tissue survival. Thus, it is considered safe to design and elevate a flap whose length approximates the distance between two perforators, without increasing the risk of distal tissue ischemia or necrosis. [24,25].

The vascular connection pattern between two adjacent perforators plays a critical role in determining the extent to which tissue can be safely elevated beyond the second perforator. This relationship is governed by the nature of the anastomotic vessels linking the corresponding perforasomes. Two primary types of anastomoses have been described in the literature: true anastomoses and choke anastomoses. True anastomoses are characterized by consistent vessel caliber and blood flow between adjacent perforasomes, effectively allowing for immediate and robust perfusion across perforator boundaries. These connections behave as a single hemodynamic unit, enabling more liberal flap elevation with minimal concern for distal ischemia [25]. In contrast, choke vessels are smaller-caliber vascular channels that function as regulatory gateways between neighboring vascular territories. These vessels typically exhibit a physiological narrowing and are capable of modulating blood flow between perforasomes in a delayed or adaptive manner. The hemodynamic resistance within choke anastomoses may initially limit perfusion beyond the second perforator; however, over time, these vessels can undergo dilation or remodeling in response to surgical manipulation or increased metabolic demand [26]. Understanding the balance between true and choke anastomoses is essential for predicting flap viability, particularly when designing extended or sequentially based perforator flaps. Consequently, choke vessels enable the harvesting of greater perforator flaps such as multiterritory perforator flaps, which integrate surrounding perforasomes into a flap initially based on a single main perforator [27].

Perforator mapping is critical in flap design, especially in anatomically constrained or compromised vascular territories such as the lower limb. Our findings revealed that IRT had a sensitivity of 97.4% and a positive predictive value (PPV) of 96.8%, outperforming D-US in sensitivity, though D-US maintained a slightly higher PPV. This is consistent with previously published data suggesting the utility of IRT in real-time perforator visualization [28]. For example, in a study regarding abdominal perforator mapping, Orădan et al. observed a sensitivity of 95.27% while using IRT, higher than for D-US [29]. Another study by Meier et al. shows that 92.3% of hotspots on the anterolateral thigh correspond to perforators localized in a 3 cm radius, showcasing IRT’s benefit in preoperative perforator mapping and flap planning [30].

Moreover, the alcohol-based cooling and rewarming stress test further improved detection accuracy by enhancing the contrast between active perforators and surrounding tissue—a technique supported by prior studies, which used cooling modalities to provoke reactive hyperemia, thereby making “hot spots” more visible [18,31,32].

The positive correlation between the number of detected perforators and flap success is strongly supported in the literature. Our study found statistically significant differences between the number of perforators detected in flaps with and without complications, a finding echoed by authors such as Saint-Cyr et al., who emphasized the role of clustered perforators and perforasome theory in optimizing perfusion [33]. Moreover, Gupta et al. demonstrated that the risk of ischemic adverse effects and necrosis in propeller flaps is correlated with the number and quality of the perforators [34].

Flap geometry played a notable role in predicting complications. Our data suggested that, in V-Y and Keystone flaps, lower A/C and B/C ratios (indicative of smaller flaps relative to the defect) were associated with additional complications. This occurrence is probably explained by tension-induced ischemia, leading to flap vascular insufficiency, as described by Gao et al. [35]. Conversely, in propeller flaps, larger B/C ratios led to more complications, potentially due to excessive rotation angles or increased flap size relying on a single perforator territory, which has been previously associated with increased risk of flap failure [36].

IRT proved effective in the early identification of flap distress, demonstrating a high capacity of predicting complications by postoperative day seven. The assessment of absolute temperature, as well as temperature gradients, informs the surgeon about areas that demand close surveillance and particular treatment. These results align with prior literature indicating that thermal asymmetry and hypothermia in a flap may precede clinical signs of vascular compromise. For instance, Singla et al. found that flaps with surface temperatures >1 °C lower than surrounding skin had higher ischemic failure rates, with mean temperature differences of 2.42 °C registered in complicated cases [37]. Furthermore, Chava et al. observed that flaps suffering from vascular injury had a temperature gradient of 2.4 °C or greater one day after surgery [19]. Our data confirm that a day 7 gradient >1.19 °C is a meaningful predictor of complications, making IRT a useful non-invasive monitoring tool. Furthermore, the large effect size (Cohen’s d = 1.84) associated with postoperative flap temperature readings indicates a robust predictive value.

Diabetes mellitus (DM) is known to have a deleterious effect on microvascular perfusion and wound healing. Chronic hyperglycemia, hypertension, and dyslipidemia induce metabolic and hemodynamic changes that fuel inflammation, endothelial dysfunction, oxidative stress, and fibrosis [38]. The presence of diabetes mellitus emerged as a significant predictor of postoperative complications in our study group, with all nine complicated cases suffering from DM, and a mean complication rate of 37% for diabetic patients. A study by Chen et al. reported similar complication rates for diabetic patients, ranging from 27.27% to 47.37% [39]. Moreover, data analysis showed that elevated preoperative glycemia (>180 mg/dL) was significantly associated with an increased risk of wound dehiscence and minor flap necrosis, corroborating findings from Chen et al., who noted higher rates of perioperative complications in the hyperglycemic population [40].

Tobacco consumption is known for its detrimental vascular effects through peripheral vasoconstriction and hindered oxygen release from hemoglobin. Tobacco use lowers nitric oxide (NO) bioavailability, resulting in increased oxidative stress in the vasculature. NO is an endogenous molecule responsible for a variety of key endothelial functions, including vasodilation, anti-thrombotic, and anti-inflammatory effects. Oxidative stress represents a significant factor in the inflammatory response and is now recognized as an important early component in the development of atherosclerosis [41]. The smoking effect on flap survival has been in discussion among experts for a long time, with conflicting conclusions. Some authors, such as Ooms et al., observe an insignificant influence of tobacco consumption on flap survival rates [42]. On the other hand, Booi et al. showed considerable differences in postoperative results between smokers and non-smokers [43]. Our data showed lower flap temperatures and higher temperature gradients for tobacco consumers, especially at postoperative day 7. While not statistically significant, this observation supports the vasoconstrictive and endothelial-damaging effects of nicotine and carbon monoxide.

The integration of IRT into clinical workflows offers several benefits: improved flap design accuracy, reduced intraoperative uncertainty, and earlier detection of complications. Given the non-contact, radiation-free, and real-time nature of IRT, it can be seamlessly integrated into perioperative protocols with minimal cost and training requirements.

Despite its strengths, the study has limitations. The sample size, while respectable, restricts subgroup analysis, particularly in assessing the nuanced interplay of systemic and flap-specific factors. Larger, multicenter trials could enhance the statistical power and generalizability of the findings. Furthermore, the range of systemic factors whose impact was analysed was rather limited, and we admit that other factors may also influence flap outcomes. Parameters such as body mass index, chronic renal disease, cardiovascular comorbidities, including obliterating angiopathy of the lower limbs and chronic venous insufficiency, were not evaluated in our study, although they could potentially influence not only the thermographic measurements but also the flap’s healing process and its vulnerability to infection and ischemia. Another limitation is the subjective interpretation of thermal images, which introduces inter-observer variability. Future developments in AI-assisted thermogram analysis may provide more standardized, automated assessment tools, reducing variability and increasing clinical applicability.

## 5. Conclusions

The results of this study underscore the utility of IRT as a powerful predictive tool, complementing preoperative planning and postoperative monitoring of perforator-based flaps used in lower limb reconstruction. By combining thermal imaging with Doppler ultrasound (D-US) validation, this study demonstrates a high level of accuracy in perforator detection, as well as a significant correlation between thermal readings and flap viability. Moreover, it also identifies key systemic and flap design-related risk factors for postoperative complications. Thus, IRT demonstrates significant utility as a fast and non-invasive modality for intraoperative and postoperative perfusion assessment in flap surgery. Its ability to provide immediate thermographic mapping improves perforator selection accuracy and enables early detection of ischemia alterations, resulting in enhanced flap viability and surgical results.

## Figures and Tables

**Table 1:** Flap dimensions depending on flap type.

	V-Y Flap			Keystone Flap			Propeller Flap		
	Min.	Max.	Mean	Min.	Max.	Mean	Min.	Max.	Mean
Flap length (cm) = A	7.4	19.5	12.6	7.8	20.6	12.8	10.8	23.4	16.1
Perforator–flap edge distance (cm) = B	4.0	8.7	6.2	4.4	9.8	6.9	7.5	14.3	10.9
Perforator–defect edge distance (cm) = C	6.9	18.8	11.7	7.4	18.0	12.5	6.9	13.9	10.3

**Table 2:** Flap ratios and impact on complications.

		V-Y Flaps			Keystone Flaps			Propeller Flaps		
	Complications	Mean	Std. Dev.	*p*-Value	Mean	Std. Dev.	*p*-Value	Mean	Std. Dev.	*p*-Value
B/C	No	0.552	0.059	0.318	0.549	0.058	0.073	1.053	0.030	0.037
	Yes	0.476	0.063		0.627	0.044		1.087	0.032	
A/C	No	1.092	0.095	0.079	1.017	0.101	0.498	1.552	0.089	0.063
	Yes	0.962	0.110		0.963	0.177		1.638	0.078	

**Table 3:** Number of detected perforators.

		Number of Cases by Detection Method		
		IRT	D-US	Intraoperatively
Number of perforators detected	1	19	26	21
	2	36	35	33
	3	18	14	19
	4	3	1	3

**Table 4:** Correlation between perforators and flap complications.

	Complications	Mean	Std. Dev.	*p*-Value
Thermal perforators	No	2.13	0.796	0.042
	Yes	1.56	0.726	
Doppler perforators	No	1.94	0.736	0.022
	Yes	1.33	0.707	
Intraoperative perforators	No	2.15	0.803	0.005
	Yes	1.33	0.707	

**Table 5:** Impact of diabetes and smoking on flap outcome.

		Complications			
		No	Yes	*p*-Value	Phi
Diabetes Mellitus	No	52	0	*p* < 0.001	0.539
	Yes	15	9		
Smoker status	No	36	2	0.076	0.276
	Yes	31	7		

**Table 6:** Influence of paraclinical parameters on flap outcome.

	Complications	Mean	Std. Dev.	*p*-Value	Cohen D
CRP (mg/L)	No	16.71	28.30	0.065	−1.18
	Yes	54.36	52.45		
Hb (g/dL)	No	13.29	1.76	0.972	0.00
	Yes	13.31	1.54		
Glycemia (mg/dL)	No	116.42	48.32	0.05	−1.22
	Yes	181.22	83.50		
WBC (×10^3^/μL)	No	8.34	3.54	0.157	−0.51
	Yes	10.14	3.73		

**Table 7:** Mean flap temperatures depending on diabetic status.

	Diabetes	Mean	Std. Dev.	*p*-Value
Flap temperature on day one (°C)	No	33.9	0.76	0.044
	Yes	33.5	0.82	
Flap temperature on day seven (°C)	No	35.8	0.35	<0.001
	Yes	35	0.90	
Temperature difference on day one (°C)	No	3.06	0.52	0.991
	Yes	3.07	0.71	
Temperature difference on day seven (°C)	No	0.40	0.25	<0.001
	Yes	0.98	0.55	

**Table 8:** Mean flap temperatures depending on smoker status.

	**Smoker Status**	**Mean**	**Std. Dev.**	***p*** **-Value**
Flap temperature on day one (°C)	No	34.1	0.75	<0.001
	Yes	33.5	0.73	
Flap temperature on day seven (°C)	No	35.9	0.33	<0.001
	Yes	35.3	0.81	
Temperature difference on day one (°C)	No	3.01	0.55	0.396
	Yes	3.12	0.61	
Temperature difference on day seven (°C)	No	0.39	0.24	<0.001
	Yes	0.77	0.53	

**Table 9:** Influence of flap temperature on complications.

	Complications	Mean	Std. Deviation	*p*-Value	Cohen’s D
Flap temperature on day one (°C)	No	33.8	0.81	0.023	0.51
	Yes	33.4	0.46		
Flap temperature on day seven (°C)	No	35.7	0.52	0.015	1.84
	Yes	34.6	1.04		
Temperature difference on day one (°C)	No	3.05	0.60	0.375	−0.33
	Yes	3.23	0.37		
Temperature difference on day seven (°C)	No	0.51	0.36	0.015	−1.69
	Yes	1.19	0.66		

## Data Availability

The raw data supporting the conclusions of this article will be made available by the authors on request.

## Abbreviations

The following abbreviations are used in this manuscript:

IRT	Infrared Thermography
D-US	Doppler Ultrasonography
DM	Diabetes Mellitus
NO	Nitric Oxide
CRP	C-reactive Protein
WBCs	White Blood Cells
PPV	Positive Predictive Value
